# Centiloid values from deep learning-based CT parcellation: a valid alternative to freesurfer

**DOI:** 10.1186/s13195-025-01860-1

**Published:** 2025-09-30

**Authors:** Yeo Jun Yoon, Seungbeom Seo, Sangwon Lee, Hyunkeong Lim, Kyobin Choo, Daesung Kim, Hyunkyung Han, Minjae So, Hosung Kang, Seongjin Kang, Dongwoo Kim, Young-gun Lee, Dongho Shin, Tae Joo Jeon, Mijin Yun

**Affiliations:** 1 Yonsei University College of Medicine, 50-1 Yonsei-ro, Seodaemun-gu, Seoul, 03722 Republic of Korea; 2 Department of Nuclear Medicine, Yonsei University College of Medicine, 50-1 Yonsei-ro, Seodaemun-gu, Seoul, 03722 Republic of Korea; 3 Department of Computer Science, Yonsei University, 50 Yonsei-ro, Seodaemun-gu, Seoul, 03722 Republic of Korea; 4 Department of Artificial Intelligence, Yonsei University, 50 Yonsei-ro, Seodaemun-gu, Seoul, 03722 Republic of Korea; 5 ELTEC College of Engineering, Ewha Woman’s University, 52 Ewhayeodae- gil, Seodaemun-gu, Seoul, 03760 Republic of Korea; 6 Department of Nuclear Medicine, Hallym University Sacred Heart Hospital, 22 Gwanpyeong-ro, Dongan-gu, Anyang, 14068 Republic of Korea; 7 Department of Neurology, Ilsan Paik Hospital, Inje University College of Medicine, 170 Juhwa-ro, Ilsanseo-gu, Goyang, 10380 Republic of Korea; 8 Massachusetts College of Pharmacy & Health Sciences, Boston, USA; 9 Department of Nuclear Medicine, Gangnam Severance Hospital, Yonsei University College of Medicine, 211 Eonjuro, Gangnam-gu, Seoul, 06273 Republic of Korea

**Keywords:** Alzheimer’s disease, Centiloid, Florbetaben, Amyloid imaging

## Abstract

**Background:**

Amyloid PET/CT is essential for quantifying amyloid-beta (Aβ) deposition in Alzheimer’s disease (AD), with the Centiloid (CL) scale standardizing measurements across imaging centers. However, MRI-based CL pipelines face challenges: high cost, contraindications, and patient burden. To address these challenges, we developed a deep learning-based CT parcellation pipeline calibrated to the standard CL scale using CT images from PET/CT scans and evaluated its performance relative to standard pipelines.

**Methods:**

A total of 306 participants (23 young controls [YCs] and 283 patients) underwent 18 F-florbetaben (FBB) PET/CT and MRI. Based on visual assessment, 207 patients were classified as Aβ-positive and 76 as Aβ-negative. PET images were processed using the CT parcellation pipeline and compared to FreeSurfer (FS) and standard pipelines. Agreement was assessed via regression analyses. Effect size, variance, and ROC analyses were used to compare pipelines and determine the optimal CL threshold relative to visual Aβ assessment.

**Results:**

The CT parcellation showed high concordance with the FS and provided reliable CL quantification (R² = 0.99). Both pipelines demonstrated similar variance in YCs and effect sizes between YCs and ADCI. ROC analyses confirmed comparable accuracy and similar CL thresholds, supporting CT parcellation as a viable MRI-free alternative.

**Conclusions:**

Our findings indicate that the CT parcellation pipeline achieves a level of accuracy similar to FS in CL quantification, demonstrating its reliability as an MRI-free alternative. In PET/CT, CT and PET are acquired sequentially within the same session on a shared bed and headrest, which helps maintain consistent positioning and adequate spatial alignment, reducing registration errors and supporting more reliable and precise quantification.

**Supplementary Information:**

The online version contains supplementary material available at 10.1186/s13195-025-01860-1.

## Background

Amyloid positron emission tomography / computed tomography (PET/CT) imaging has emerged as a pivotal tool for quantifying amyloid-beta (Aβ) deposition in vivo, with significant implications for understanding Alzheimer’s disease (AD) pathology [1]. This quantification enables the early detection of Aβ plaques and facilitates tracking their progression over time, which is critical for diagnosis, prognosis, and monitoring treatment effects [2, 3]. The importance of accurately quantifying Aβ burden has become even more pronounced with the emergence of monoclonal antibodies such as lecanemab and donanemab, which target aggregated Aβ to reduce its accumulation in the brain [4, 5]. However, variability in tracers, imaging protocols, and analysis methods hampers cross-center comparisons and meta-analyses. This variability also complicates the establishment of universal diagnostic thresholds, highlighting the critical need for standardized approaches to ensure consistency and broader applicability in clinical and research settings.

The Centiloid (CL) project was initiated to standardize the quantification of Aβ burden across PET/CT imaging centers, addressing variability in tracers and analysis methods [6]. By establishing a standardized pipeline for cortical standardized uptake value ratios (SUVRs) and enabling cross-calibration of different tracers, this approach integrates data onto a unified scale. This framework facilitates comparisons across studies and enhances the utility of amyloid PET imaging in both clinical and research settings.

Traditional PET quantification has relied on magnetic resonance imaging (MRI) as an anatomical reference, but this requirement presents several challenges. MRI may be unavailable due to cost, patient burden, or contraindications such as claustrophobia or medical implants, potentially excluding certain participants and introducing selection bias [7, 8]. To address these limitations, several studies have explored MRI-free quantification methods, utilizing PET-based or CT-based approaches by developing standardized templates and aligning PET images with these templates for quantification [9, 10]. Previous studies have noted that template-based methods may face limitations in patients with significant brain atrophy, including reduced spatial accuracy and increased susceptibility to partial volume effects, particularly when spatial normalization fails to account for severe anatomical distortions [8, 11].

To address these issues, several studies have attempted CT-based segmentation using deep learning models trained on SPM-derived tissue labels, direct application of SynthSeg to CT, or CT-to-MR translation followed by MR segmentation. However, these approaches either lack fine anatomical detail, are prone to segmentation failures, or require multi-step pipelines [12–14]. In contrast, a recently developed deep learning-based (DL-based) CT parcellation method provides a robust alternative to traditional MRI-based approaches by directly segmenting CT images according to Desikan-Killiany-Tourville (DKT) atlas, thereby overcoming the limitations of previous methods [15]. This DL-based approach, which employs three independent 2D UNet-based segmentation models to capture multi-view anatomical features, provides accurate definitions of cortical gyri and maintains reliable quantification even in severely deformed brains, such as those with atrophy or hydrocephalus. The acquisition of PET and CT in the same session using a shared scanner bed and headrest helps maintain spatial alignment, thereby reducing errors and improving reliability. This approach overcomes the limitations of MRI-free methods while remaining accessible and cost-effective, making amyloid PET quantification feasible even for individuals unable to undergo MRI. It may serve as a useful alternative when MR-based processing is not feasible and anatomical variation is a concern.

In this study, we implemented a DL-based CT parcellation pipeline calibrated to the standard CL scale using CT images from PET/CT scans and determined a cutoff value based on agreement with visual reads. We then evaluated its performance by comparing it with CL scales derived from previously established pipelines, assessing agreement with visual reads, and comparing effect sizes between AD and non-AD groups.

## Methods

### Participants

We recruited 23 young controls (YCs) and 283 patients who presented to our memory clinic with memory complaints and underwent ^18^F-florbetaben (FBB) PET/CT and MRI between January 2017 and December 2022. YCs, defined as those younger than 45 years, had no history of neurologic or psychiatric disorders, confirmed normal cognitive function after a clinical evaluation, and were Aβ-negative on PET imaging. Of the 283 patients, 207 were Aβ-positive and 76 were Aβ-negative based on visual assessment. In the Aβ-negative patients (*n* = 76), 70 patients had amnestic mild cognitive impairment (aMCI), and 6 patients had dementia. Among Aβ-positive patients (*n* = 207), 55 patients had AD dementia, and 152 patients had aMCI. Patients who were Aβ-positive and diagnosed with either dementia or aMCI were collectively referred to as the AD-related cognitive impairment (ADCI) group. AD dementia was diagnosed based on the National Institute on Aging-Alzheimer’s Association criteria for probable AD [16]. Participants with aMCI met the criteria proposed by Petersen et al. [17]: (1) subjective memory complaints, (2) relatively normal performance in other cognitive domains, (3) normal activities of daily living (ADL), (4) objective memory impairment below − 1.5 SD on either verbal or visual memory tests, and (5) not demented. The following exclusion criteria were applied to the 283 patients: (1) presence of structural brain lesions – such as brain tumors, multiple lacunar infarcts, or cerebral infarctions on MRI – and (2) a time gap of over one year between the FBB PET/CT scans and MRI.

### Image acquisition

FBB PET/CT scans were acquired using the Discovery 600 system (GE Healthcare, Milwaukee, WI). A total of 300 MBq of FBB was administered intravenously, and PET imaging was performed 90 min post-injection for 20 min, followed by a CT scan for attenuation correction. The CT scan was performed in spiral mode with acquisition parameters of 0.8 s per rotation, 120 kVp, 200 mA, a slice thickness of 3.27 mm, a collimation of 10 mm, and a table feed of 9.375 mm per rotation. The PET images (matrix size: 256 × 256, voxel size: 0.98 mm × 0.98 mm × 3.27 mm) were reconstructed using the ordered subset expectation maximization (OSEM) algorithm with 4 iterations and 32 subsets, incorporating attenuation, scatter, and random corrections. A Gaussian smoothing filter with a full-width at half-maximum (FWHM) of 4 mm was applied to the reconstructed images. Non-contrast T1-weighted MRI scans were obtained on a 3-T MRI system (Ingenia CX or Achieva; Philips Healthcare, Best, Netherlands) with a matrix of 256 × 256, a field of view ranging from 230 to 240 mm, a slice thickness of 1.2 mm, and a repetition time/echo time (TR/TE) of 6.9/3.2 milliseconds.

### Visual assessment

Visual assessment of FBB PET/CT scans was performed by two expert nuclear medicine physicians (M. Yun and T.J. Jeon), both blinded to the amyloid PET quantification results. The evaluation employed a standardized approach based on a regional cortical tracer uptake (RCTU) scoring system applied to four brain regions including lateral temporal cortex, frontal cortex, posterior cingulate cortex/precuneus, and parietal cortex. The RCTU assessments were then integrated into the overall brain amyloid plaque load (BAPL) score, leading to a binary classification of the scans [18, 19]. A BAPL score of 1 (no Aβ load) is classified as an Aβ-negative PET scan, while BAPL scores of 2 (minor Aβ load) and 3 (significant Aβ load) are classified as Aβ-positive PET scans.

### Data processing - Centiloid pipeline

All PET and MRI images were processed using an implementation adapted from the standard CL pipeline with SPM 12, based on procedures described in [5]. In brief, each subject’s MRI image was segmented and normalized to MNI space using SPM12, and the PET image was co-registered to the corresponding MRI and subsequently normalized using the same transformation parameters. The standard cortical and whole cerebellum reference regions of interest (ROIs) were downloaded from the GAAIN website (www.gaain.org). Finally, we calculated the CL pipeline-derived SUVR (SUVR_standard_) in MNI space using the standard cortical target region and the whole cerebellum as reference region.

### Local pipeline validation procedures

As described in section 2.2.2 of Klunk [6], the first step of a Level 2 analysis begins with a replication of the Level-1 analysis. To validate our local pipeline, we downloaded the reference ^11^C-Pittsburgh Compound-B (PiB) PET (50–70 min) dataset from GAAIN. Briefly, this dataset includes 34 young controls (YC) and 45 older adults with clinically diagnosed AD, which serve as the CL scale’s anchor points of 0 and 100 units, respectively. We processed the downloaded dataset using our local pipeline, yielding local Level-1 outcomes. We then compared these local CL values against published CL values. All processing was performed using SPM12 (version 12; https://www.fil.ion.ucl.ac.uk/spm/software/spm12/).

### Data processing - FreeSurfer pipeline

We analyzed MRIs using FreeSurfer (FS) v7.4 to generate a native-space FS atlas for each MRI (http://surfer.nmr.mgh.harvard.edu). We subsequently co-registered FBB PET images to their corresponding MRIs with SPM12’s “Coregister: Estimate and Reslice” tool using default parameters. We then sampled the PET images to assess the mean tracer uptake in target cortical areas using a cortical mask made up of FS-defined frontal, cingulate, lateral parietal, and lateral temporal regions as previously described [20]. We then assessed the mean tracer uptake in four reference regions: the whole cerebellum (WC), cerebellar gray matter (CG), pons, and a composite reference region. The composite reference region was created by taking the unweighted average of FS-defined whole cerebellum, brainstem, and eroded subcortical white matter, following previously established methods [21, 22]. The eroded subcortical white matter mask was generated by first smoothing a binarized FS-defined subcortical white matter image to the 8mm^3^ resolution of the FBB PET image, followed by thresholding at 0.70 to selectively erode white matter-defining voxels near gray matter. Using mean tracer uptake in the cortical target areas and these reference regions, we calculated FS pipeline-derived SUVRs (SUVR_FS_) for each FBB PET scan.

### Data processing - CT parcellation pipeline

We performed CT-based FBB quantification using our previously developed DL-based CT parcellation method, utilizing commercially available software (NCM-brain v2.5, Newcure M, Seoul, Korea). Briefly, the pipeline employs three independent 2D U-Net segmentation networks—one each for axial, sagittal, and coronal CT slices—each configured with 256 feature channels per layer to accommodate CT’s lower soft-tissue contrast [15]. The outputs of these three models are ensembled to generate a complete 1 mm isotropic 3D brain parcellation, which is then co-registered to PET space. PET images were sampled using the same cortical mask as in the FS pipeline to calculate mean tracer uptake in the target cortical areas. We used the same four reference regions employed in the FS pipeline – WC, CG, pons, and the composite reference region. Using the mean tracer uptake in the cortical target areas and these reference regions, we calculated CT parcellation pipeline-derived SUVRs (SUVR_CT_) for each FBB PET scan. The co-registration process was omitted as FBB and CT images were acquired with consistent positioning in a single PET/CT session, allowing adequate alignment.

### Centiloid conversion

We performed a regression on the corresponding SUVR_FS_ and SUVR_CT_ values. From these equations, we determined the intercept (b) and slope (m), which were then used to convert SUVR_CT_ into SUVR_FS_. The conversion equations for SUVR_FS_ to CL, specifically for WC and composite reference region, have already been published [20]. Using these equations, the “calculated” SUVR_FS_ were subsequently converted into CL units.

For the standard CL pipeline, the FBB tracer is a well-established surrogate tracer for PiB, and the conversion equation from the CL pipeline-derived FBB-SUVR to PiB-SUVR has been validated in previous studies [23]. Thus, the FBB-SUVR_standard_ values were subsequently converted into “calculated” PiB-SUVR_standard_ values. Using Eq. 2.2.3 from Klunk et al. [6] and substituting our Level-1 YC-0 and AD-100 CL values, these “calculated” PiB-SUVR_standard_ values were converted into CL units.

$$\begin{array}{l}\:{\rm{CL}} = 100\: \times \:\left( {{\rm{PiB - SUV}}{{\rm{R}}_{standard}}\: - \:1.012} \right)/\\\:(2.077\: - \:1.012)\end{array}$$

The standard CL pipeline uses WC as the reference region and generates a single CL value per patient, abbreviated as CL_standard_. In contrast, the FS and CT parcellation pipelines, each of which uses four reference regions, produce CL values abbreviated as CL_FS_ and CL_CT_, respectively. Specifically, the FS pipeline produces the following values: CL_FS−WC_ (WC), CL_FS−CG_ (CG), CL_FS−pons_ (pons), and CL_FS−comp_ (composite reference region); and for the CT parcellation pipeline: CL_CT−WC_ (WC), CL_CT−CG_ (CG), CL_CT−pons_ (pons), and CL_CT−comp_ (composite reference region).

### Statistical analysis

Demographic characteristics, including both continuous variables and ordinal variables, were compared using the Kruskal-Wallis test, with post-hoc comparisons conducted using Dunn’s test with Bonferroni correction. For two-group comparisons, the Wilcoxon rank-sum test was used. To compare values against zero, the Wilcoxon signed-rank test was performed. Correlations between SUVR values were assessed using linear regression analysis. The effect size between the ADCI and YC groups was calculated with the following equation:

$$\begin{array}{l}{\rm{Effect}}\:{\rm{Size}}\:{\rm{ = }}\left( {\mu {\:_p} - \mu {\:_n}} \right)/\\\sqrt {\left( {{N_p}\sigma \:_p^2 + {N_n}\sigma \:_n^2} \right)/\left( {{N_p} + {N_n} - 2} \right)} \end{array}$$

where $\:{{\upmu\:}}_{p}$, $\:{{\upmu\:}}_{n}$ are the average SUVR in the ADCI and YC groups. $\:{{\upsigma\:}}_{p}^{2}$ ,$\:{{\upsigma\:}}_{n}^{2}$ are the variance of the ADCI and YC groups. *N*_*p*_ and *N*_*n*_ are the number of participants in the ADCI and YC groups. Effect sizes are computed separately for each reference region within the same pipeline, and their 95% confidence intervals (CIs) were estimated using non-parametric bootstrapping with 10,000 resamples. Differences in effect sizes were considered statistically significant when the corresponding CIs did not overlap.

Within the YC group, we evaluated the variance of each imaging pipeline and reference. First, Levene’s test was performed to assess homogeneity of variance among CL values obtained using WC – CL_standard_, CL_CT−WC_, and CL_FS−WC_. When significant differences were detected, post-hoc pairwise F-tests were conducted. Next, pairwise F-tests were performed to compare the variances between the two reference regions within each pipeline (CL_CT−WC_ versus CL_CT−comp_ and CL_FS−WC_ versus CL_FS−comp_). *P*-values for all pairwise comparisons were adjusted using the Bonferroni correction for multiple comparisons.

Receiver operating characteristic (ROC) curve analysis was performed to determine the CL cutoff that showed the highest agreement with the visual read. Youden’s J index was calculated as the sum of sensitivity and specificity minus one, and the threshold maximizing this index was selected as the optimal cutoff. Classification performance was further evaluated using accuracy, sensitivity, specificity, and the area under the ROC curve (AUC). All statistical analyses were performed using the R software (version 4.0, http://www.r-project.org).

## Results

### Demographics

The baseline demographics and clinical characteristics of the participants are summarized in Table 1. There was a significant difference in age between the YC group and both the Aβ-negative and ADCI groups. The Korean version of the Mini-Mental State Examination (K-MMSE) scores were highest in the order YC, Aβ-negative, and ADCI, with significant differences among all three groups. In contrast, Clinical Dementia Rating (CDR) global scores were highest in ADCI, followed by Aβ-negative and then YC, with significant differences observed across the three groups.

**Table 1:** Demographics and clinical characteristics of the participants

	YC	Aβ-negative	ADCI	*p*-value
*N*	23	76	207	N/A
Age, years ^a, b^	30.3 ± 7.98	71.8 ± 7.70	72.0 ± 8.18	< 0.001
Females, n (%)	14 (61%)	61 (80.3%)	138 (66.7%)	0.054
K-MMSE ^a, b, c^	29.8 ± 0.491	23.8 ± 4.19	21.9 ± 4.39	< 0.001
CDR global score ^a, b, c^				< 0.001
0	23	0	0	
0.5	0	70	151	
1	0	5	45	
≥ 2	0	1	11	

Note: Values are presented as number (%) for categorical variables, mean ± SD for continuous variables, and median (IQR) for ordinal variables (CDR score)

Abbreviations: YC, young control; Aβ, amyloid beta; ADCI, Alzheimer’s disease-related cognitive impairment; K-MMSE, Korean version of the Mini-Mental State Examination; CDR, Clinical Dementia Rating

^a^ Significant differences between YC and Aβ-negative

^b^ Significant differences between YC and ADCI

^c^ Significant differences between Aβ-negative and ADCI

### Local pipeline validation

**Fig. 1:**
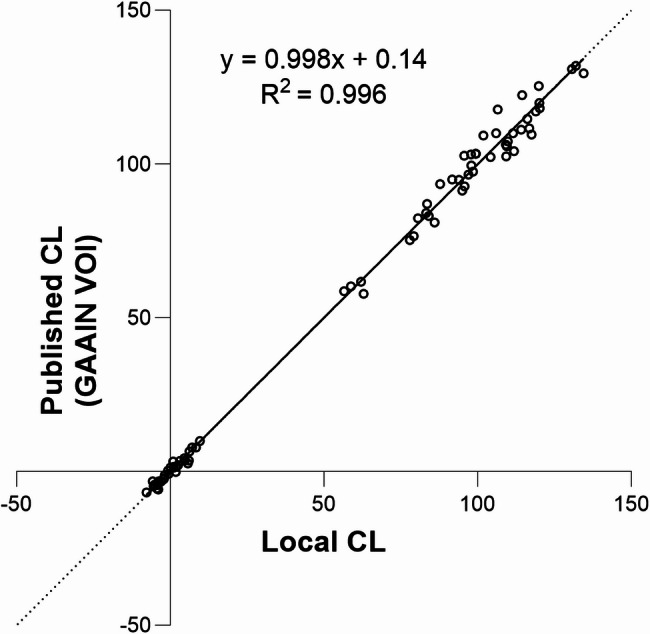
Plot of CL outcomes derived from Level-1 analysis of the standard 34 YC-0 and 45 AD-100 scans vs. published CL values. Dashed unity lines have been added to facilitate visual comparison between the axes. The equation and R^2^ indicate that the local Centiloid pipeline was applied correctly. Abbreviation: CL, Centiloid; VOI: volume of interest

Linear regression of the local standard CL level 1 outcomes against published CL outcomes yielded a fit equation with slope = 0.998, intercept = 0.140, and correlation coefficient (R^2^) = 0.996 (Fig. 1). The fit exceeded the minimum specified acceptance criteria (i.e., R^2^ > 0.98, slope between 0.98 and 1.02, and intercept between − 2 and + 2), confirming that the local results derived from SPM12 were comparable with published CL from GAAIN.

### FreeSurfer versus CT parcellation pipeline

**Fig. 2:**
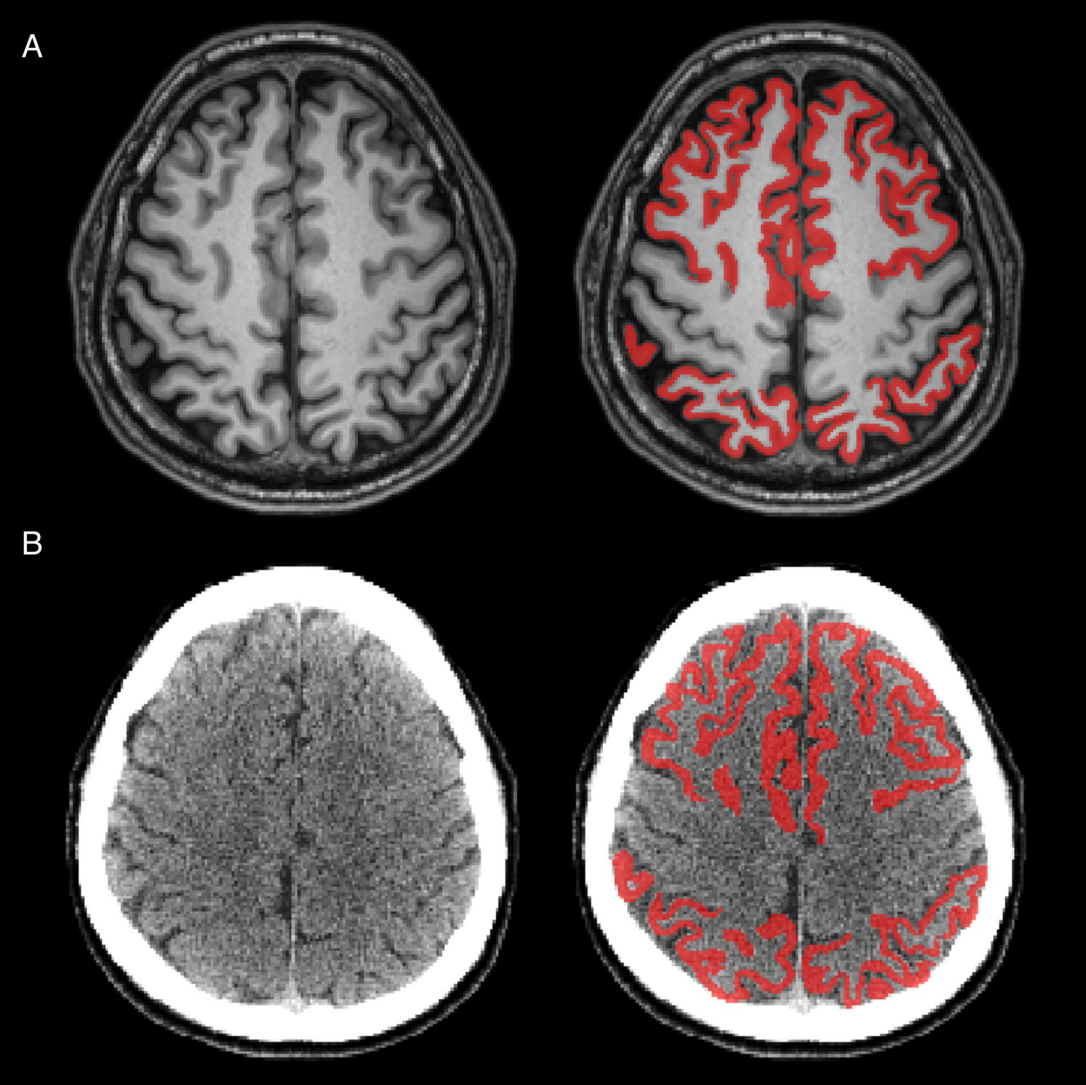
Cortical mask overlaid on original MRI and CT images. (**A**) Representative images of MRI after segmentation using the FS pipeline. Left: original MRI scan; Right: same scan with the cortical mask overlaid, delineating the frontal, cingulate, lateral parietal, and lateral temporal regions. (**B**) Representative CT images following segmentation using the CT parcellation pipeline. Left: original CT scan; Right: same scan with the cortical mask overlaid, delineating the corresponding target regions. Abbreviation: FS, FreeSurfer

**Fig. 3:**
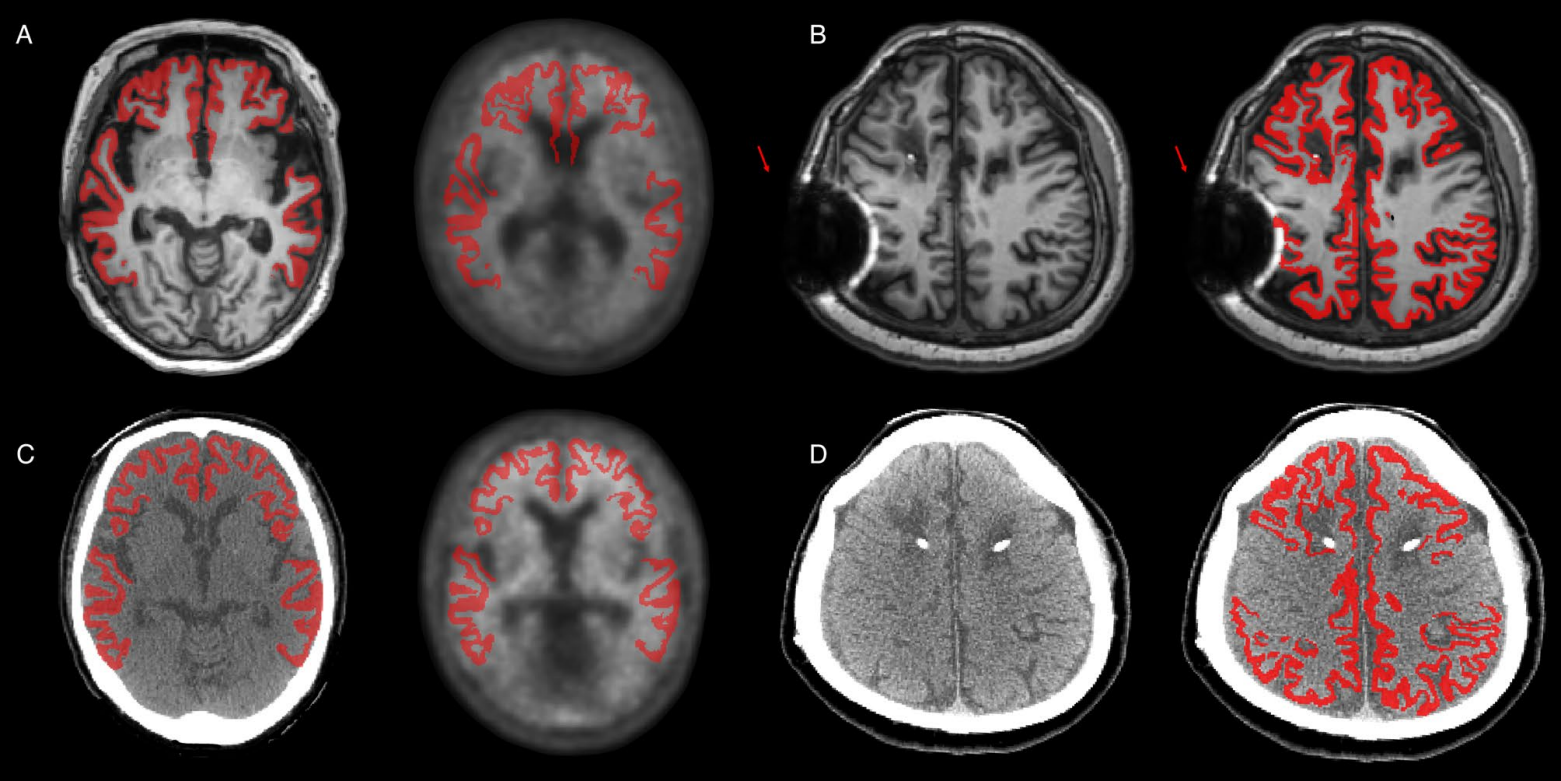
Representative cases that were excluded from regression analysis. (**A**) The outlier case from the FS pipeline, showing suboptimal co-registration between MRI and PET/CT. Left: original MRI with the FS-derived cortical mask; Right: co-registered PET/CT image in which the mask does not align properly. (**B**) Case with a pronounced susceptibility artifact on MRI. Left: original MRI displaying the artifact; Right: same MRI with the FS-based cortical mask applied. (**C**) Corresponding CT images for the outlier in (**A**). Left: original CT scan; Right: CT parcellation-derived cortical mask overlaid on the co-registered PET/CT image. (**D**) Corresponding CT image for the artifact case in (**B**). Left: original CT scan; Right: CT parcellation-derived cortical mask overlaid on the PET/CT image. Both cases illustrated in (**A**) and (**B**) were excluded from the regression analysis. Abbreviation: FS, FreeSurfer

**Fig. 4:**
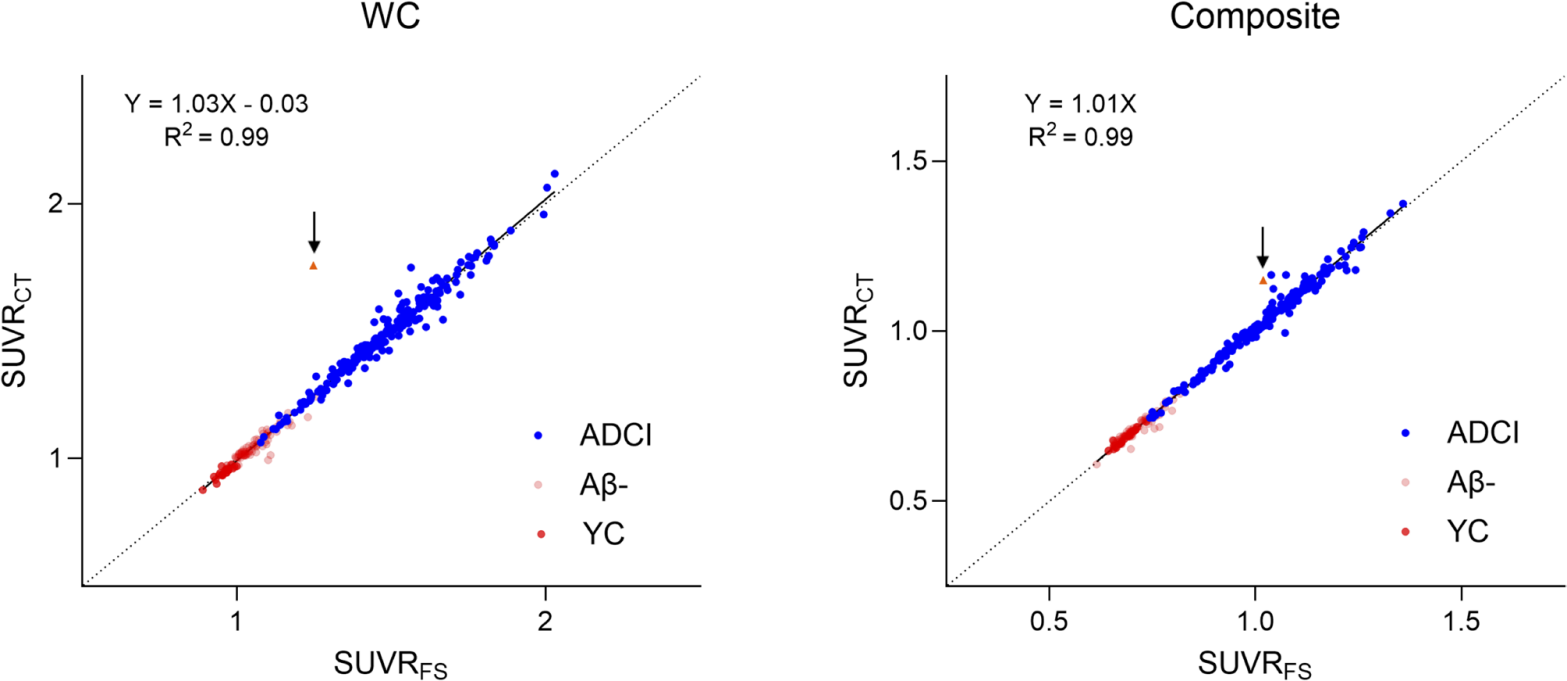
Linear regression of SUVR_FS_ against SUVR_CT_ for the whole cerebellum (WC) and the composite reference region. The scatter plots display the relationship between SUVR_FS_ and SUVR_CT_ for each reference region. Dashed unity lines have been added to facilitate visual comparison between the axes. An outlier with inaccurate co-registration (as shown in Fig. 3A and C) is indicated by an orange triangle and black arrow in the scatter plot. Abbreviation: FS, FreeSurfer; SUVR_FS_, FS pipeline-derived standardized uptake value ratio; SUVR_CT_, CT parcellation pipeline-derived standardized uptake value ratio; WC, whole cerebellum; Composite, composite reference region

MRI and CT images were segmented using the FS and CT parcellation pipelines. Both pipelines demonstrated accurate anatomical segmentation, allowing reliable ROI-based quantification (Fig. 2). However, in one case, a susceptibility artifact on MRI resulted in inaccurate segmentation by FS; in another case, inaccurate co-registration between MRI and PET led to unreliable PET quantification; both cases were removed from further analysis (Fig. 3). Following segmentation, the cortical mask of the target regions was applied, and SUVR_CT_, SUVR_FS_ were calculated for each reference region. After plotting SUVR_FS_ and SUVR_CT_, we performed a regression analysis. Regression analysis between the FS pipeline and the CT parcellation pipeline demonstrated high concordance across all reference regions. Results for WC and composite reference regions are shown in Fig. 4, and results for CG and pons are provided in Supplementary Fig. 1.

### Conversion equations

SUVR_FS_ and SUVR_CT_ were plotted and regressed against each other for WC and the composite reference region. The slope and intercept values derived from the regression equations were used to convert SUVR_CT_ into “calculated” SUVR_FS_. Using the previously known equation described in previous work, SUVR_FS_ values were then converted into CL [20].

For the standard CL pipeline, FBB-SUVR_standard_ values were converted into “calculated” PiB-SUVR_standard_ values [23]:

$$\begin{array}{l}\:{\rm{PiB - SUV}}{{\rm{R}}_{standard}} = {\rm{(FBB - SUV}}{{\rm{R}}_{standard}}\:\\- \:0.39)/0.61\end{array}$$

PiB-SUVR_standard_ were finally converted into CL units. The final conversion equations are as summarized in Table 2.

**Table 2:** CL conversion equations with FS and CT-based pipelines for each reference region

Reference region	FS pipeline	CT parcellation pipeline	Standard CL pipeline
WC	CL = 157.15 × SUVR – 151.87	CL = 153.05 × SUVR – 143.63	CL = 154.0 × SUVR – 155.1
Composite	CL = 244.20 × SUVR – 170.80	CL = 234.01 × SUVR – 168.12	N/A

Abbreviations: CL, Centiloid; FS, FreeSurfer; SUVR, standardized uptake value ratio; SUVR, standardized uptake value ratio; WC, whole cerebellum; Composite, composite reference region

### Effect size, variability, and distribution of CL values

The variance in YC reflects the noise inherent in the quantification method and has been used to evaluate both pipelines and reference regions [6, 24]. In our study, when using WC as the reference region, both CL_FS_ and CL_CT_ consistently showed the lowest variance among YC groups (Table 3). When comparing different pipelines using WC as the reference region, no significant difference was observed between CL_FS−WC_ and CL_CT−WC_ (*p* = 0.73); however, both CL_FS−WC_ (*p* = 0.038) and CL_CT−WC_ (*p* = 0.017) exhibited significantly lower variance than CL_standard_.

**Table 3:** CL values across reference regions and groups

Reference region	CL_FS_		CL_CT_		CL_standard_
	WC	Comp	WC	Comp	WC
ADCI					
Mean	85.27	78.53	85.15	72.28	83.81
SD	28.12	28.67	27.91	27.93	30.70
YC					
Mean	0.91	-3.78	0.99	-7.73	-6.66
SD	**3.85** ^a^	5.61	**3.57** ^a^	5.25	**6.83**
Effect size	**3.15**	3.01	**3.17**	3.01	**3.10**

Abbreviations: CL, Centiloid; FS, FreeSurfer; CL_FS_, FS pipeline-derived CL value; CL_CT_, CT parcellation pipeline-derived CL value; CL_standard_, Centiloid pipeline-derived CL value; ADCI, patients with AD dementia and mild cognitive impairment; YC, young control; WC, whole cerebellum; Comp, composite reference region

^a^ Significant difference from CL_standard_

In addition, the effect size between the ADCI and YC groups was highest with WC in the FS (3.15, 95% CI: 2.62–3.66) and CT parcellation (3.17, 95% CI: 2.65–3.69) pipelines. When comparing the pipelines using WC as the reference region, the effect sizes of CL_standard_ (3.10, 95% CI: 2.58–3.61), CL_FS−WC_ (3.15, 95% CI: 2.62–3.66) and CL_CT−WC_ (3.17, 95% CI: 2.65–3.69) did not differ significantly. Overall, WC was determined to be the optimal reference region in the CT parcellation pipeline, which demonstrated performance that was similar to either FS or the standard CL pipeline.

### ROC analysis results

Both the FS and CT parcellation pipelines showed comparable performance in the ROC analysis. ROC curves were generated based on visual read outcomes. The AUC was 0.994 versus 0.995 for the FS and CT parcellation pipelines, respectively, with the optimal thresholds identified via Youden’s J index were 29.9 and 29.7. At these thresholds, FS and CT parcellation exhibited comparable performance: accuracy – 0.964 versus 0.967; sensitivity – 0.957 versus 0.957; and specificity – 0.980 versus 0.990, respectively. These results suggest that both pipelines provide equally robust classification performance for identifying Aβ-positive scans, with minimal differences in AUC, accuracy, sensitivity, and specificity.

## Discussion

In this study, we calculated the CL conversion equation for our recently developed DL-based CT parcellation method. Using the derived equation, CT parcellation pipeline-derived CL scales showed high agreement with the CL scales generated by both FS and the CL pipeline. Notably, when using WC as the reference region, CL_FS_ and CL_CT_ demonstrated high concordance, with an R² of 0.99. Furthermore, the optimal thresholds for the two pipelines were similar (29.9 for CL_FS_ and 29.7 for CL_CT_), falling within the previously reported range of 17 to 40 CL, with most studies citing values between 25 and 35 CL [9, 25–29]. This consistency reinforces the validity of our approach, and its alignment with established visual cutoffs supports the clinical potential of the CT parcellation pipeline as a viable alternative to the FS pipeline.

Early brain PET quantification relied on template-based methods, aligning PET images to a standardized framework for ROI-based analysis. MRI-free approaches using CT or PET templates have been explored but struggle with accurately defining intricate cortical structures and are prone to distortions in cases with severe atrophy or hydrocephalus [8, 11]. Parcellation-based methods, like FS, improve accuracy by extracting precise ROIs from MRI. Our CT parcellation method retains these advantages while eliminating the need for MRI, enhancing accessibility and robustness [15]. First, the CT parcellation pipeline enables rapid and cost-effective CL quantification for a broader patient population. Unlike MRI, which requires long scan times and may be inaccessible due to cost or contraindications [7, 8], CT scans are acquired simultaneously during PET/CT scans, significantly reducing acquisition time and improving accessibility. This is particularly beneficial following the introduction of monoclonal antibody therapies, as regular Aβ imaging is essential for tracking Aβ accumulation. By enabling Aβ quantification without high-resolution MRI, this approach may facilitate more frequent treatment monitoring, ultimately improving AD management. Second, in PET/CT scans, CT and PET images are acquired sequentially within the same session using a shared bed and headrest, which helps maintain consistent positioning and facilitates spatial alignment. This typically results in improved co-registration, supporting more precise quantification. In contrast, MRI and PET are susceptible to co-registration failures due to factors such as noise, limited spatial resolution, and time gaps between acquisitions. For instance, we observed a case where accurate co-registration between MRI and PET failed and resulted in inaccurate quantification (Fig. 3). Although SPM12, used in the FS pipeline, has been reported to outperform other tools such as NiftyReg and Vinci in MRI-PET co-registration accuracy, the FS pipeline is still not entirely free from co-registration issues [30].

As previously mentioned, YC variance and the effect size between ADCI and YC are key variables used in cross-sectional studies for comparing reference regions and pipelines. Klunk et al. previously investigated these two variables to identify the optimal reference region for the standard CL pipeline [6]. In this study, the YC variance of the standard CL pipeline was significantly higher than that of both the FreeSurfer and CT parcellation pipelines. Since YC variance reflects the noise inherent in a quantification method, the template-based method may be more susceptible to noise—possibly due to its inability to perform quantification along accurate cortical gyri—compared to the parcellation-based method. This increased variance might also result from the fact that the cortical ROI in the standard CL pipeline was derived from PiB PET data, which may not fully apply to FBB PET. Further studies are needed to determine the superiority between template-based and parcellation-based methods.

Biologically, the cerebellum is favorable because it is free of Aβ deposition and exhibits nondisplaceable activity similar to the target cortical area [31, 32]. Previous cross-sectional and longitudinal studies have further supported WC as the most reliable reference region for FBB [24, 33]. In parallel, recent studies on longitudinal Aβ quantification using PiB and ^18^F-Florbetapir have indicated that a composite reference region, which combines WC, brainstem, and eroded subcortical white matter, may offer improved longitudinal stability [20, 21, 34]. In this study, we compared WC and the composite reference region; although the difference was not statistically significant, WC consistently yielded better performance in terms of YC variance. This may reflect the fact that subcortical white matter is more susceptible to atrophy, vascular lesions, and signal spillover [21]. However, our data were acquired in a single-center dataset with uniform acquisition settings. In multi-center studies where scanner types and participant positioning vary, the composite reference region may offer better longitudinal consistency.

Regarding the use of CT in neurodegenerative disease screening, CT-based assessments have shown significant potential, with recent advancements in DL techniques further enhancing their capabilities. CT quantification has demonstrated performance comparable to MRI in measuring brain atrophy and white matter lesions [35]. Additionally, CT-based volumetric measures can differentiate patients with neurodegenerative diseases from healthy controls and are strongly associated with cognitive, biochemical, and neuroimaging markers [36]. Our CT parcellation-based CL approach further extends this potential by enabling quantitative amyloid PET analysis without the need for MRI, making amyloid burden assessment more accessible and cost-effective. Moreover, emerging technologies such as photon-counting CT offer high-resolution imaging with lower radiation exposure [37]. As CT resolution and soft tissue contrast continue to improve, its utility in screening not only for AD but also for other neurodegenerative diseases may increase further. Additionally, extracting more information from the CT performed simultaneously with PET could enable a more comprehensive assessment in a single scan, providing significant benefits for patients.

This study has several limitations. First, we increased the tube current to 200 mAs for high-quality parcellation, resulting in an effective dose of 0.39 mSv from CT. However, this additional dose is minimal compared to the total PET/CT effective dose, which exceeds 4 mSv [19]. Several recent studies have investigated CT image denoising techniques aimed at enabling reliable anatomical analysis from low-dose CT scans [38, 39]. Applying such denoising methods prior to parcellation may allow our current pipeline to be extended to dose-reduced CT images. In addition, recent studies have demonstrated that PET tracer doses can be substantially reduced to as low as 12.5% of the original dose without compromising quantitative accuracy in CL scaling [40]. Together, these complementary strategies may help minimize overall radiation exposure in PET/CT protocols and improve the feasibility of the proposed method in diverse research and clinical settings. Second, as this is a single-center study, validation in different centers, with various PET tracers and scanner modes, is required to establish broader applicability. Incorporating a more diverse training dataset – encompassing scans with structural abnormalities and those from multiple scanners and tracers – could improve robustness and broaden clinical applicability. Leveraging publicly available imaging datasets that include PET/CT and MR scans could enable external validation and help assess generalizability across diverse populations and acquisition protocols. Furthermore, our pipeline is designed specifically for PET/CT data and is not currently applicable to PET/MRI. Given the increasing adoption of PET/MRI scanners, extending compatibility to this modality is an important direction for future development. Third, although CT and PET are acquired sequentially using the same headrest and positioning setup, our pipeline is not entirely free from co-registration error due to potential patient movement. In addition, while the CL standard pipeline is based on SPM8, our analysis employed SPM12. Despite strong agreement in Level 1 validation, segmentation differences between versions may introduce methodological variability. Fourth, we did not directly compare our CT-based pipeline to PET-only template methods (e.g., rPOP). Although our high concordance with MRI-guided FreeSurfer supports the accuracy of CT parcellation, a head-to-head evaluation against PET-only approaches would help determine which method enables more accurate quantification. Finally, longitudinal validation of the CL scale is necessary to establish its robustness in tracking Aβ changes over time. While the CT parcellation pipeline has demonstrated strong performance in cross-sectional studies, its stability and its comparability to other pipelines for monitoring disease progression remain unclear. Further studies are required to assess whether CL_CT_ remains stable in Aβ-negative individuals and whether it can detect subtle but clinically meaningful changes in Aβ-positive individuals over time.

## Supplementary Information

Below is the link to the electronic supplementary material.

Supplementary Material 1

## Data Availability

No datasets were generated or analysed during the current study.

## Abbreviations

Aβ: Amyloid-beta
AD: Alzheimer’s disease
ADCI: Alzheimer’s disease-related cognitive impairment
ADL: Activities of daily living
aMCI: Amnestic mild cognitive impairment
AUC: Area under the ROC curve
BAPL: Brain amyloid plaque load
CDR: Clinical dementia rating
CI: Confidence interval
CL: Centiloid
MRI: Magnetic resonance imaging
FBB: ^18^F-florbetaben
FS: FreeSurfer
K-MMSE: Korean version of the Mini-Mental State Examination
PiB: ^11^C-Pittsburgh Compound-B
RCTU: Regional cortical tracer uptake
ROC: Receiver operating characteristic
ROI: Region of interest
SUVR: Standardized uptake value ratios
WC: Whole cerebellum
