# Supplementary material for: Centiloid values from deep learning-based CT parcellation: a valid alternative to freesurfer
Source: Alzheimers Res Ther. 2025 Sep 30;17:212. doi: 10.1186/s13195-025-01860-1 (PMC12482646; doi:10.1186/s13195-025-01860-1)
Supplement: Supplementary file 1 — Supplementary Material 1 [file 13195_2025_1860_MOESM1_ESM.docx]

**
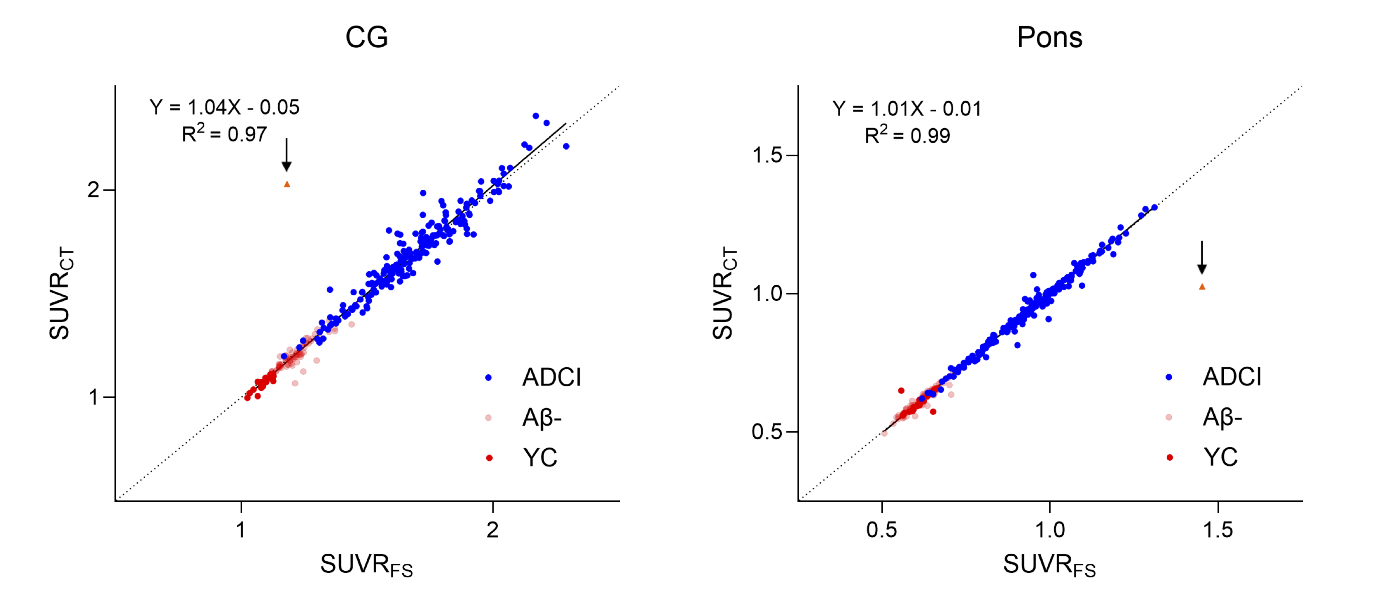
**

**Supplementary Fig. 1** Linear regression of SUVR_FS_ against SUVR_CT_ for the cerebellar gray matter (CG) and pons. The scatter plots display the relationship between SUVR_FS_ and SUVR_CT_ for each reference region. Dashed unity lines have been added to facilitate visual comparison between the axes. An outlier with inaccurate co-registration (as shown in Figure 3A and 3C) is indicated by an orange triangle and black arrow in the scatter plot. Abbreviation: FS, FreeSurfer; SUVR_FS_, FS pipeline-derived standardized uptake value ratio; SUVR_CT_, CT parcellation pipeline-derived standardized uptake value ratio; CG, cerebellar gray matter.
